# Rare case report: non-mass invasive ductal carcinoma presenting with rectal and cervical lymph node metastasis as the initial symptom

**DOI:** 10.3389/fonc.2025.1606116

**Published:** 2025-06-25

**Authors:** Qiong Zhang, Zhifeng Xiong, Yuan Gao, Min Zeng, Huachao Yang, Gang Lyu

**Affiliations:** ^1^ Department of Breast, The Third Affiliated Hospital of Beijing University of Chinese Medicine, Beijing, China; ^2^ Department of Breast, Chongqing Hospital of Traditional Chinese Medicine, Chongqing, China

**Keywords:** non-mass breast cancer, invasive ductal carcinoma, rectal metastasis, cervical lymph node metastasis, case report

## Abstract

It is uncommon for breast cancer to present with distant metastasis as the initial symptom. This study reported a 67-year-old female patient with breast non-mass invasive ductal carcinoma, who sought medical attention due to abdominal distention, lower abdominal pain, constipation, hematochezia, and left-sided neck swelling. After a thorough examination, pathology confirmed the diagnosis of breast invasive ductal carcinoma, along with cervical lymph node and rectal metastasis. The non-specific breast symptoms in this case posed challenges for the early diagnosis of breast cancer. This also suggests that for breast cancer patients without a history of gastrointestinal disease, the presence of changes in bowel habits should raise suspicion for metastatic lesions. Imaging combined with biopsy pathology plays an important role in the diagnosis and treatment of non-mass breast cancer. This case also underscores the importance of breast health awareness and routine breast cancer screening among women, both for clinical practice and public health initiatives. Notably, early identification and diagnosis of non-mass breast cancer, coupled with the development of personalized treatment plans through multidisciplinary collaboration, are essential for enhancing treatment efficacy.

## 1 Introduction

Breast cancer is one of the most commonly diagnosed cancers in women worldwide (1). The 5-year survival rate for patients with advanced breast cancer remains below 20% (2). The primary therapeutic objective in this population is to prolong survival while maintaining an acceptable quality of life. Nevertheless, distant metastasis constitutes the leading cause of breast cancer–related mortality (3), posing a significant challenge to long-term prognosis. The most common sites of distant metastasis of breast cancer include the bones, lungs, liver, and brain, while metastasis to the rectum is rare (4). In reports on gastrointestinal metastasis of breast cancer (5), the probability of gastrointestinal metastasis in invasive lobular carcinoma (ILC) is 4.5%, while for invasive ductal carcinoma, it is only 1.1%. In terms of lymph node metastasis, the axillary and supraclavicular lymph nodes are common sites of metastasis in breast cancer, while cervical lymph node metastasis, especially submental lymph node metastasis is rarely reported. We reported a rare case in which the patient presented with abdominal distension, lower abdominal pain, constipation, hematochezia, and cervical swelling. Pathological examination confirmed metastatic lesions in the rectum and cervical lymph nodes, both originating from the breast. However, the patient had no obvious breast mass, and the clinical presentation was consistent with non-mass breast cancer, which led to delayed diagnosis and treatment.

Breast lump is one of the common symptoms of breast cancer. Non-mass breast cancer usually lacks obvious lumps, and the early symptoms are atypical and easily overlooked. Early manifestations, including minor changes in breast skin, spontaneous nipple discharge, or enlargement of axillary lymph nodes, may constitute the only detectable clinical signs and are often misinterpreted or ignored. In this case, the patient lacked awareness of breast health and did not have enough knowledge of abnormal body changes, which was one of the major reasons for the delay. A comprehensive physical examination ultimately led to the detection of lesions involving both the breast and rectum. This case highlights the indispensable value of comprehensive physical examination in the diagnostic process of non-mass breast cancer. As a vital adjunct to imaging and histopathological evaluation, physical examination continues to play a crucial role in enhancing diagnostic precision and facilitating timely clinical intervention.

This case exhibited distinct characteristics in terms of metastatic sites, breast cancer subtypes, and clinical presentations. Our report provided a comprehensive summary of the clinical manifestations, diagnosis, treatment, and potential metastatic mechanisms of this rare case, intending to offer guidance for the diagnosis and management of breast cancer. Additionally, this case also highlights the importance of women’s self-breast health awareness and breast cancer screening programs for the medical community and the public.

## 2 Case presentation

A 67-year-old Chinese female presented in June 2024 with abdominal distension after meals, lower abdominal pain, constipation, hematochezia without any obvious cause,along with swelling in the left cervical region and a palpable mass in the submandibular area.The patient had no history of gastrointestinal or head and neck disorders. Gastrointestinal endoscopy at local hospitals was performed, revealing multiple polyps in the colon and rectum, treated with argon plasma coagulation. The patient was preliminary diagnosed with “chronic gastritis, chronic rectitis, chronic colitis, colon polyps, rectal polyps, and Helicobacter pylori infection.” Due to limited medical resources, the local hospital provided only symptomatic treatment for Helicobacter pylori infection. The patient was prescribed “Esomeprazole Magnesium Enteric-coated Tablets (20 mg once daily), Amoxicillin (1 g twice daily), Clarithromycin (0.5g twice daily), and Bismuth Potassium Citrate capsules (110 mg four times daily),” resulting in slight symptom improvement.The pathological examination of the rectal biopsy tissue at a later stage revealed irregular nests of atypical cells, suggesting the possibility of poorly differentiated carcinoma. It was suggested that the patient seek further consultation at a higher-level hospital.

Upon presentation to a higher-level hospital, the patient underwent a thorough physical examination. Examination revealed left-sided cervical swelling with overlying skin thickening and decreased elasticity, accompanied by a palpable submandibular mass. The left axillary lymph node was about 2.5 cm x 2.0 cm in size with unclear borders, restricted mobility,no obvious tenderness, and a firm texture. Further examination revealed that the patient had a flaky thickening of the glands near the areola of the left breast. Digital rectal examination was performed due to the patient’s lower abdominal pain,constipation, and hematochezia. Digital rectal examination revealed a circumferential, irregular mass approximately 5 cm from the anus. The mass was hard in texture, and the intestinal lumen was narrowed, preventing passage of the fingertip. A small amount of dark red blood was noted on the rectal examination glove. Given these abnormal findings on physical examination, further imaging studies were undertaken to delineate the lesions.

**Figure 1:**
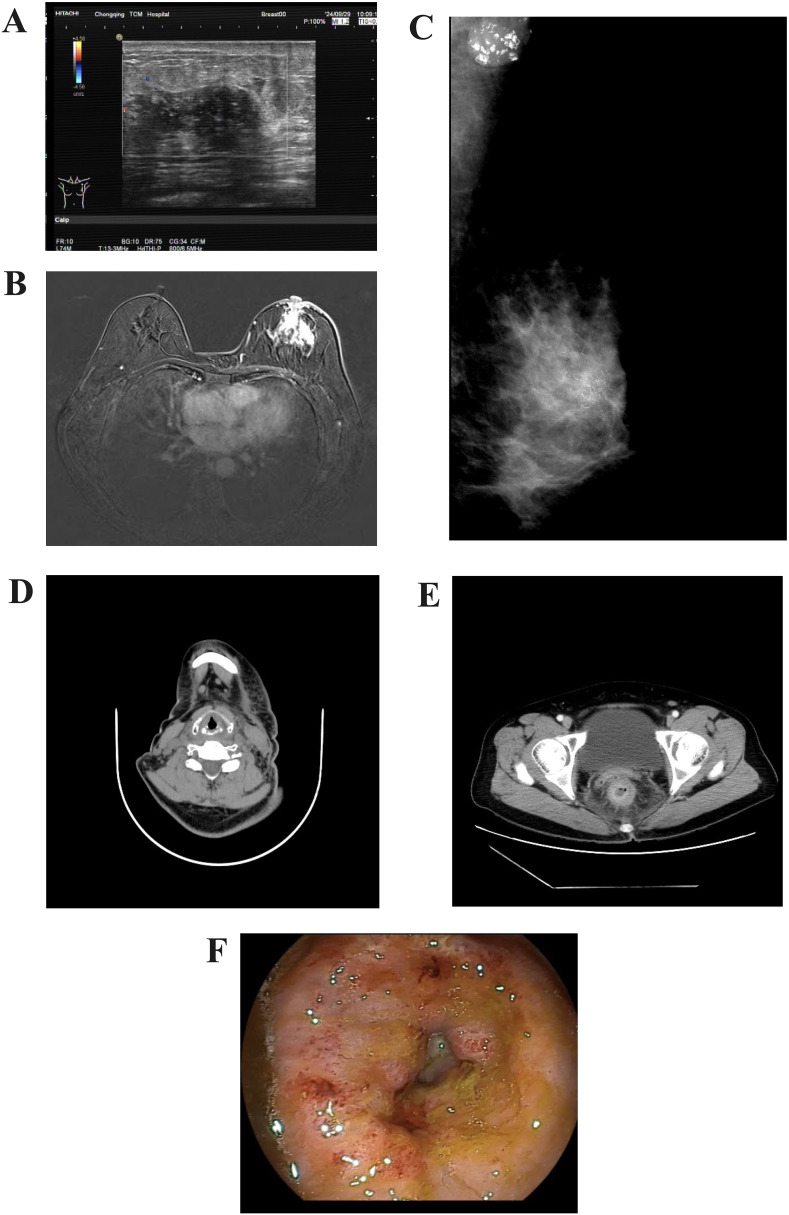
Imaging of the tumour. **(A)** Color Doppler ultrasound of the breast. **(B)** Contrast-enhanced MRI scan of the breast. **(C)** Mammography of the breast. **(D)** CT imaging of the cervical lymph nodes. **(E)** Contrast-enhanced CT scan of the lower abdomen. **(F)** Endoscopic appearance of the rectal lesion before treatment.

Breast ultrasound revealed a heterogeneous hypoechoic area within the glandular layer adjacent to the left breast areola, measuring approximately 27mm × 18mm × 9mm, with unclear boundaries, irregular margins, heterogeneous internal echoes, and increased peripheral blood flow signals (**Figure 1A**). Chest MRI with contrast enhancement revealed multiple patchy and nodular abnormal signals in the left breast, slight thickening of the left breast skin, and multiple lymph nodes in the left axillary region (**Figure 1B**). Mammography of both breasts showed a suspicious mass with multiple calcifications in the upper portion of the left breast, along with increased density in the left axillary region, where multiple calcifications were also observed (**Figure 1C**). Contrast-enhanced CT scans revealed an abnormal enhanced focus in the central area of the left breast, with enlargement of left axillary and cervical lymph nodes (**Figure 1D**). The rectal wall exhibited circumferential thickening with enhancement, a rough serosal surface, and mild blurring of surrounding fat spaces (**Figure 1E**). Several mesenteric lymph nodes were observed, some mildly enlarged.Colonoscopy demonstrated a ring-shaped mass about 5 cm from the anus, with an uneven surface, erosion, fragile consistency, and bleeding upon palpation, leading to a narrowed intestinal lumen that obstructed the passage of the endoscope (**Figure 1F**).

**Figure 2:**
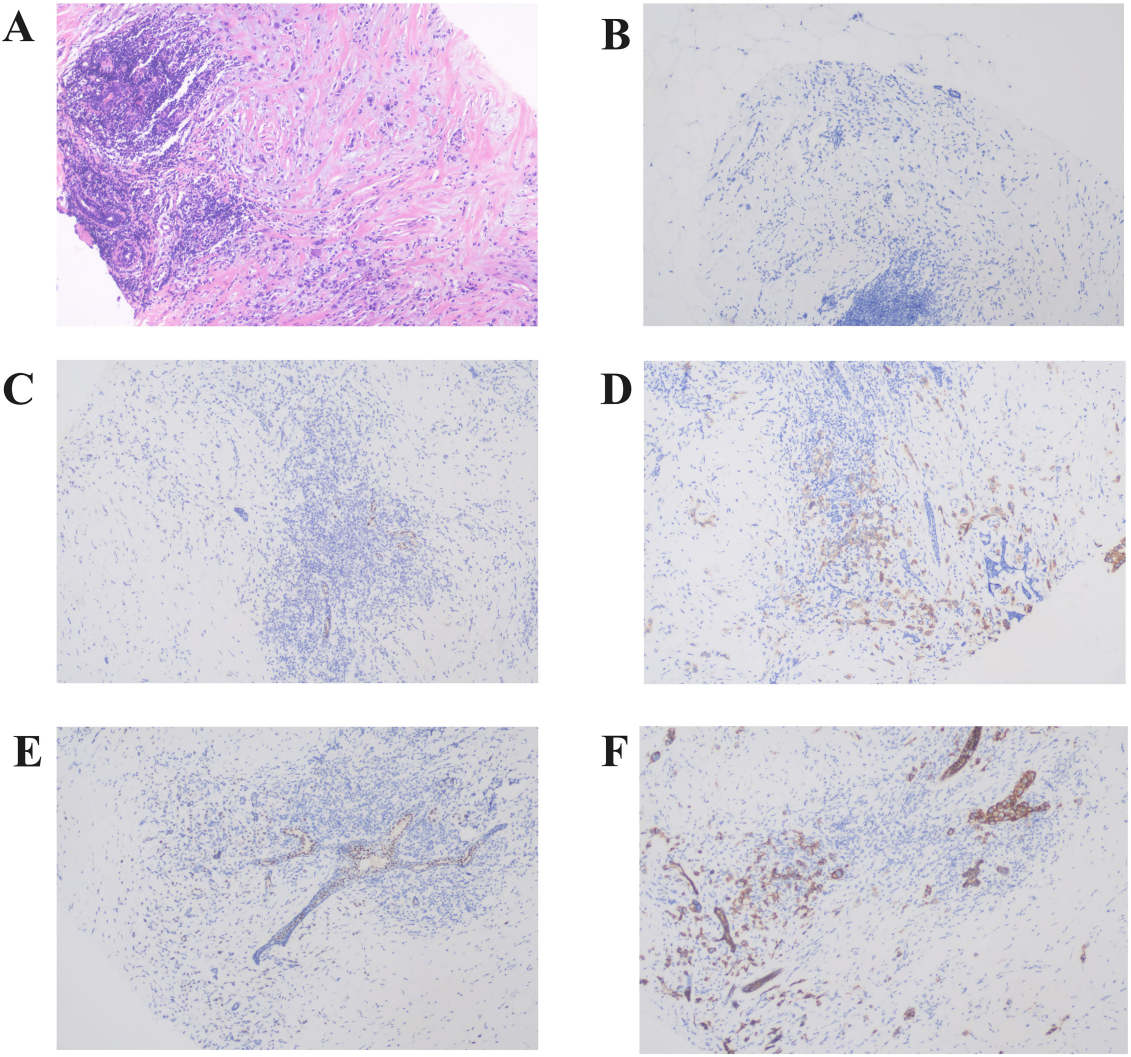
Representative histopathological findings of the left breast primary lesion. **(A)** The left breast core biopsy section showed solitary cord-like cancer cells in the periductal stroma, exhibiting invasive growth (Hematoxylin and Eosin staining,×100). **(B–F)** Immunohistochemistry revealed that the primary lesion showed negative expression of ER **(B)** and PR **(C)**, HER-2 (2+) **(D)**, positive expression of TRPS1 **(E)**, and positive expression of E-Cadherin **(F)** (×100).

Pathological diagnosis of the left breast mass by core needle biopsy revealed invasive carcinoma of no special type, grade II. Immunohistochemistry showed AR (+ approximately 60%), ER (-), PR (-), HER-2 (2+), CK5/6 (+), P63 (-), SMMHC (-), Calponin (-), TRPS1 (+), E-Cadherin (+), P120 membrane (+), Ki-67 proliferation index (+ approximately 30%), CK (+), P53 (+ approximately 90%). Fluorescence *in situ* hybridization (FISH) analysis indicated HER-2 gene amplification (**Figure 2**).

**Figure 3:**
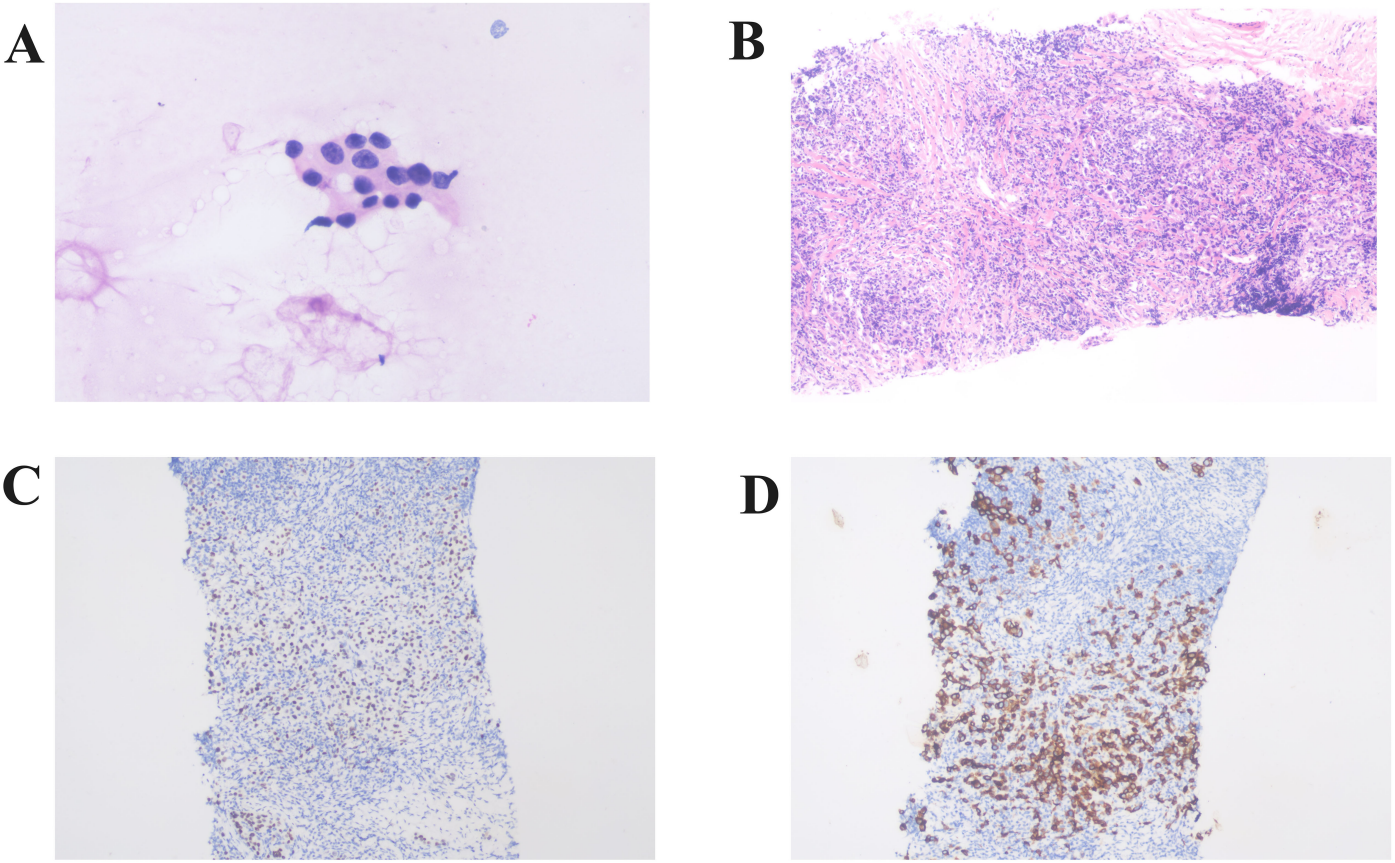
Histopathologic analysis of lymph node biopsies. **(A)** Histopathologic analysis of a submental lymph node biopsy showing malignant cells of suspected epithelial origin (×400). **(B)** Histopathological examination of the left axillary lymph node biopsy revealed the presence of carcinoma tissue, likely of breast origin (Hematoxylin and Eosin staining,×100). Immunohistochemistry shows positive expression of TRPS1 **(C)** and CK **(D)** (×100).

Fine needle aspiration of the submental lymph node revealed malignant tumor cells, suggesting an epithelial origin. Core needle biopsy of the left axillary lymph node identified cancerous tissue, likely of breast origin. Immunohistochemistry showed TRPS1 (+) and CK (+) (**Figure 3**).

**Figure 4:**
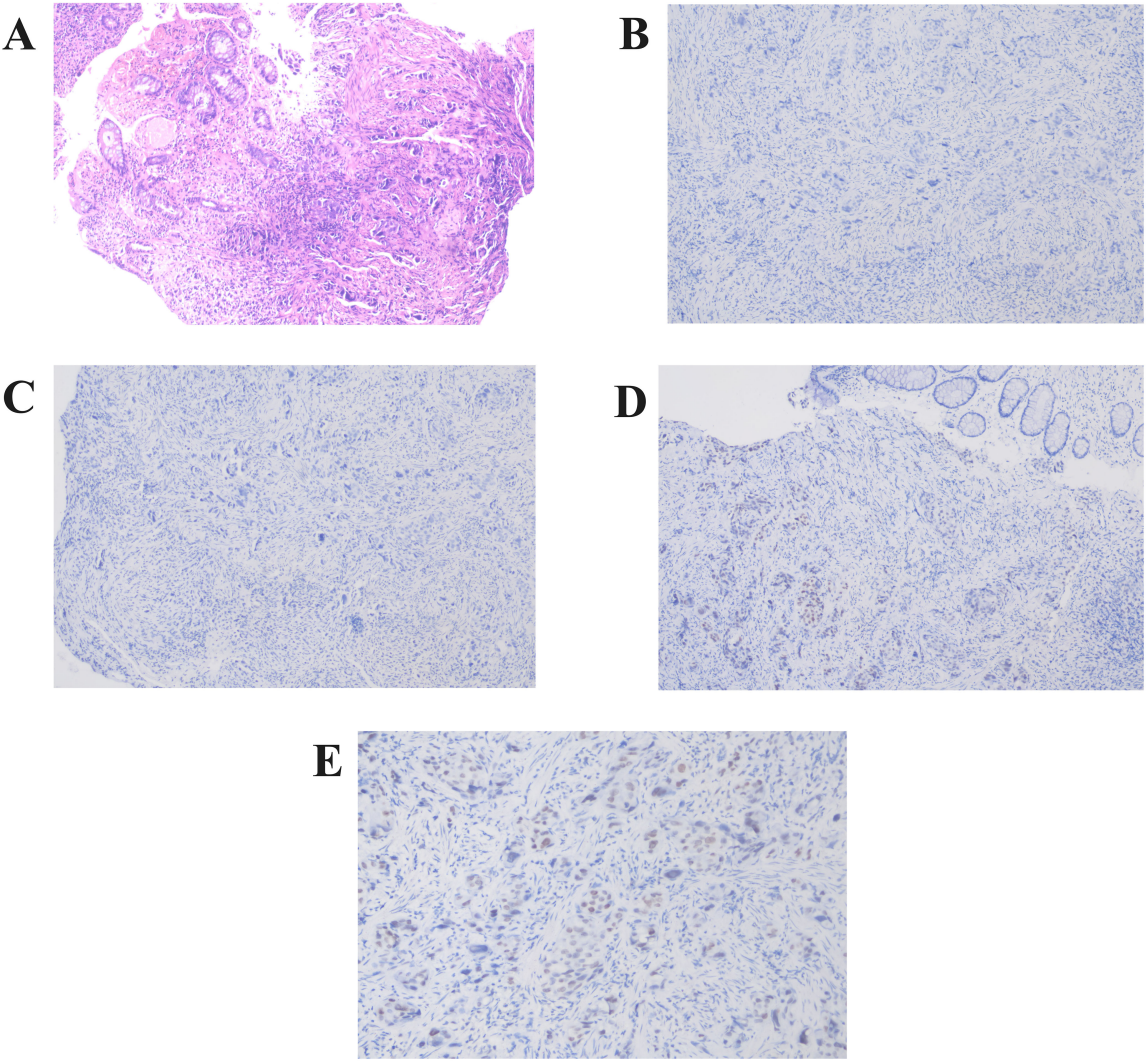
Pathological findings from colonoscopic biopsy. **(A)** Rectal wall tissue section reveals solitary, cord-like malignant tumor cells within the mucosal and muscular layers (Hematoxylin and Eosin staining,×100)). **(B–E)** Immunohistochemistry showed negative expression of CDX-2 **(B)** and SATB2 **(C)**, while GATA-3 **(D)** and TRPS1 **(E)** were positively expressed (×200).

Pathologic diagnosis from colonoscopic biopsy and immunohistochemistry confirmed breast metastasis. Immunohistochemistry exhibited CK (+), EMA (+), GATA-3 (+), TRPS1 (+), CDX-2 (-), SATB2 (-), ER (-), Ki67 (15%+) (**Figure 4**).

The patient was diagnosed with invasive ductal carcinoma of the left breast, cT3N3M1, stage IV, HER-2 positive (HR negative), with metastasis to the left axillary and cervical lymph nodes, as well as rectal metastasis. This was a case of advanced breast cancer with a poor prognosis.

After discussion in the tumor board, and based on the Cancer Society of China Oncology guidelines, the patient’s physical condition was comprehensively assessed. Due to severe gastrointestinal reactions, chemotherapy was not administered after communication with the patient and her family. The patient was subsequently treated with trastuzumab and pertuzumab (HP) as targeted therapy, administered in 21-day cycles. The specific medications administered were as follows: Trastuzumab injection, with first dose at 8 mg/kg * 56 kg = 440 mg, and subsequent doses at 6 mg/kg * 56 kg = 330 mg; and Pertuzumab injection, with the first dose at 840 mg, followed by 420 mg for subsequent doses.

**Figure 5:**
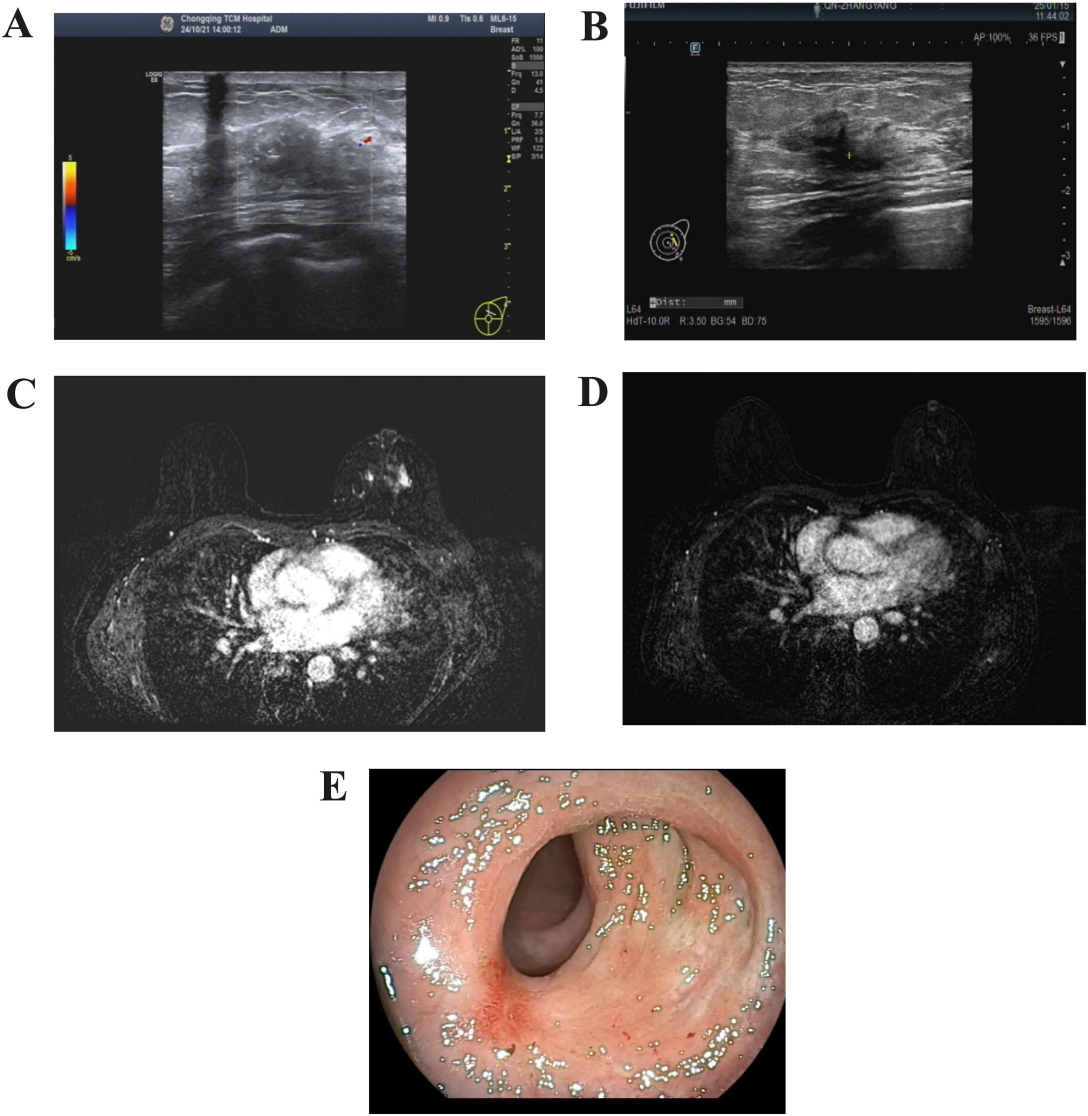
Post-treatment tumor imaging. **(A)** Color Doppler ultrasound of the breast(two cycles of HP-targeted therapy). **(B)** Color Doppler ultrasound of the breast(six cycles of HP-targeted therapy). **(C)** Contrast-enhanced MRI scan of the breast(two cycles of HP-targeted therapy). **(D)** Contrast-enhanced MRI scan of the breast(six cycles of HP-targeted therapy). **(E)** Endoscopic appearance of the rectal lesion after treatment.

Following two cycles of HP-targeted therapy, breast ultrasound and MRI assessed the left breast lesion to be reduced in size (**Figures 5A, C**), and the treatment response was evaluated as partial response (PR). Considering the patient’s overall physical condition, the addition of chemotherapy with the TCb regimen (albumin-bound paclitaxel plus carboplatin) was recommended, with the primary objective of reducing metastatic burden and prolonging survival. However, the patient declined chemotherapy due to concerns about gastrointestinal toxicity, alopecia, and other physical and psychological adverse effects. In terms of surgery, the tumor board and surgical oncology team determined that the primary indication for surgery in cases of rectal metastasis from breast cancer is palliative in nature, with the intention of improving the patient’s quality of life rather than achieving curative outcomes. At the time of assessment, the patient demonstrated regression of rectal metastatic lesions and marked improvement in symptoms, including abdominal pain and constipation. A comprehensive evaluation indicated that surgical intervention would offer limited clinical benefit under these circumstances. As a result, continuation of HP-targeted therapy was recommended as the preferred approach for subsequent treatment.

The patient underwent six cycles of HP-targeted therapy. After the treatment, abdominal distension resolved, abdominal pain was alleviated, constipation improved, and cervical swelling diminished. Imaging showed a reduction in the size of the left breast lesion (**Figures 5B, D**), and colonoscopy revealed no significant mass approximately 5 cm from the anus (**Figure 5E**).

The clinical symptom relief and the reduction in the size of the lesions observed on imaging both indicated the effectiveness of the current treatment regimen. The patient exhibited no adverse effects from the targeted therapy, suggesting good tolerance. During the treatment, the patient experienced a weight loss of 5 kg.

## 3 Discussion

Breast cancer metastasis to the rectum is rare. Reports indicate that the most common gastrointestinal metastatic sites for breast cancer are the upper digestive tract and stomach, followed by the small intestine and colon, while rectal metastasis is uncommon (6). Bolzacchini E identified 96 cases of intestinal metastasis from breast cancer (7), with 59 patients (61.4%) diagnosed with intestinal involvement after the breast cancer diagnosis, and 20 patients (20.8%) diagnosed at the same time. We investigated the largest cohort of colorectal metastatic breast cancer patients reported in the literature. This literature (8) involved a systematic review of individual patient data from case reports, small case series, and real-world cohorts, with a total of 762 patients. The median time from breast cancer diagnosis to the occurrence of colorectal metastasis was 67 months. In the literature cohort study, 37 patients (17.9%) presented with colorectal metastasis as the first sign of breast cancer. In the real-world cohort study, 29 patients (6.7%) had colorectal metastasis as the initial manifestation of breast cancer. The median overall survival after colorectal metastasis was 20.6 months. Based on these findings, colorectal metastasis has been considered an advanced event in breast cancer patients.

Metastatic breast cancer to the rectum shares clinical symptoms with primary rectal tumors, such as nodular elevations, ulcers, and polyps on endoscopy (9). Due to the lack of specificity, endoscopic differential diagnosis is challenging and requires confirmation by pathological examination.

Cytokeratin 7 (CK7) is a basic keratin primarily expressed in glandular and transitional epithelia. It is a key signaling molecule involved in regulating cell differentiation and apoptosis. CK7 is positively expressed in the mammary epithelium, endometrium, ovaries, lungs, and mesothelial cells. However, it is generally absent in the normal mucosa of the colon and rectum (10), and its lack of expression is considered strong evidence to exclude primary colorectal cancer. GATA binding protein 3 (GATA3) belongs to the GATA transcription factor family and is found exclusively in breast and urothelial carcinomas. It regulates the genetic differentiation of various cell types, participating in the growth of T cells and the development of organs such as the breast. GATA3 not only regulates ductal formation and alveolar differentiation but also plays a crucial role in the differentiation of mammary luminal epithelial cells (11). Trichorhinophalangeal syndrome type 1(TRPS1), also known as transcriptional repressor GATA-binding protein 1, is an emerging biomarker. As a marker with high expression in breast cancer, TRPS1 shows diffuse and consistent positivity in 93.2% of breast cancer cases. In contrast, its expression is absent or very low in several other malignancies, making it one of the specific diagnostic tools for breast cancer (12, 13). Caudal type homeobox genes 2 (CDX2) is a tumor suppressor gene that is specifically expressed in intestinal epithelial tumors. It plays a critical role in guiding and maintaining the morphology and function of intestinal epithelium, regulating the expression of numerous intestinal-specific genes. CDX2 is closely associated with the development of colorectal adenocarcinoma. It is expressed in over 80% of colorectal cancer tissues (14). The relatively specific expression of CDX2 in gastrointestinal tumors makes it a useful marker for distinguishing the primary site of the tumor (15). Abnormal expression of special AT-rich sequence-binding protein 2 (SATB2) has been associated with the development and progression of several cancers, including esophageal cancer, colorectal cancer, and gastric cancer (16). SATB2 is negative in most upper gastrointestinal and primary ovarian tumors, both benign and malignant. Its expression demonstrates a sensitivity of 79% and specificity of 95% for diagnosing lower gastrointestinal tract tumors (17).

Based on rectal biopsy immunohistochemistry, CK (+), CDX-2 (-), and SATB2 (-) can largely exclude tumors originating in the rectum, while GATA-3 (+) and TRPS1 (+) strongly support a breast origin. Therefore, the patient in this case was confirmed to have rectal metastasis from breast cancer.

The mechanism of rectal metastasis from breast cancer typically involves several consecutive stages: local invasion and migration from the surrounding tissues of the primary tumor; invasion into blood or lymphatic vessels; survival as circulating tumor cells (CTCs) in the bloodstream; extravasation from the circulatory system; adaptation to the microenvironment in the form of disseminated tumor cells; and finally, transformation into cells that initiate metastasis, ultimately leading to the formation of large metastatic lesions (18). The preferential organ-specific metastasis of breast cancer varies according to its molecular subtypes. The organotropism of breast cancer metastasis is regulated by the breast cancer subtype, the distinct genetic characteristics of metastatic tumor cells, and the signaling pathways involved (19). While the molecular mechanisms of breast cancer metastasis to the brain, lungs, bones, and liver have been extensively studied, research on the mechanisms underlying the rare metastasis of breast cancer to the rectum remains limited. The organotropism of breast cancer metastasis warrants further exploration, requiring large-scale genomic analyses to identify genes significantly altered in specific metastatic sites. Investigating organ-specific markers and genomic changes related to organ affinity in distant organ metastasis will help uncover the genetic background and potential pathogenic mechanisms of this case, ultimately aiding in the discovery of more effective targeted therapies to inhibit metastasis and providing valuable insights for scientific diagnosis and treatment.

Another noteworthy aspect of this case is the metastasis to the region I cervical lymph nodes (submental lymph node). In clinical practice, cervical lymph node metastasis is most commonly associated with head and neck malignancies, and there are no reports in the literature regarding metastasis to region I cervical lymph nodes caused by breast cancer. Generally, the lymphatic metastasis pathways of breast cancer mainly include the following pathways: 1. Lateral metastasis pathway: Breast cancer cells spread via the lymphatic vessels to the axillary lymph nodes, which is the primary pathway for lymphatic metastasis, draining 50% to 75% of the lymph from the breast. 2. Medial metastasis pathway: Breast cancer cells metastasize to the parasternal lymph nodes, or the lymph nodes around the internal mammary or thoracic arteries, accounting for 25% to 50% of the lymphatic drainage of breast. 3. Contralateral metastasis pathway: Breast cancer can spread from one breast to the contralateral breast and axilla through a network of fine lymphatic vessels in the chest wall skin. 4. Supraclavicular lymph node metastasis: Cancer cells can migrate directly or retrogradely to the supraclavicular lymph nodes via the lymphatic filtration of the axillary apex or internal mammary lymph nodes.

We screened the patient for head and neck-related tumors but found no evidence of lesions in the head and neck, thus ruling out the possibility of lymph node metastasis originating from head and neck malignancies. A biopsy of the submental lymph node indicated the presence of metastatic lesions, suspected to be of epithelial origin. Notably, this case represented the first reported instance of breast cancer metastasis to the submental lymph nodes. The patient also exhibited swelling on the left side of the neck, with thickened skin and reduced elasticity, which may be associated with local lymphatic obstruction due to lymph node metastasis.

There are several reported cases of rectal metastasis from lobular breast cancer, which is more prone to metastasize to rare sites compared to ductal breast cancer (20). This is attributed to the lack of E-cadherin expression in lobular breast cancer, as the loss of E-cadherin may reduce cell adhesion, facilitating invasion and metastasis, thus making lobular carcinoma more likely to migrate and settle in distant locations. This explains the rarity of rectal metastasis from ductal breast cancer in this case.

The human epidermal growth factor receptor 2 (HER-2) gene is an oncogene involved in the growth and progression of tumors, and it has become an important indicator for assessing the prognosis and guiding the treatment of malignant tumors, such as breast cancer and gastric cancer (21). Some studies have reported that HER-2 positivity is associated with the location of primary colorectal tumors, particularly in distal colorectal cancers (22). However, research on the role of HER-2 in the malignant progression and metastasis of colorectal cancer is limited and controversial. There is no data suggesting that HER-2 positive breast cancer is more prone to rectal metastasis. Nevertheless, reports have shown that molecular subtypes with high HER-2 expression tend to present as non-mass lesions (23), which is consistent with this case.

How to detect and diagnose non-mass breast cancer? It is well-known that most breast tumors are mass-type, and the majority of women can detect a lump through self-exam. However, non-mass breast cancers tend to disguise themselves and act as “silent killers.” Non-mass breast cancers often lack typical clinical manifestations, and the lesions observed in the imaging frequently present with an “ill-defined, diffuse, and without clear occupying effect” appearance, which can easily be confused with inflammatory breast lesions or other breast diseases, often leading to missed or incorrect diagnoses. This is also why the patient in this case was first diagnosed with breast cancer only after developing rectal metastasis. The survival rate of breast cancer varies according to the stage at diagnosis. The 5-year relative survival rates are > 99% for stage I, 93% for stage II, 75% for stage III, and 29% for stage IV (24). Therefore, delayed diagnosis and treatment of non-mass breast cancer is a significant cause of reduced patient survival rates.

Non-mass breast cancer accounts for 9.21% of breast abnormalities (25). This subtype of breast cancer does not exhibit significant spatial occupying effects in various directions, lacks typical imaging characteristics, and currently lacks a standardized imaging diagnostic criterion. The X-ray mammographic features of non-mass breast cancer mainly include four manifestations: clustered or grouped microcalcifications, localized glandular structural distortion, focal asymmetry of glandular density, and diffuse or localized increased glandular density. The typical ultrasound features are hypoechoic areas in a sheet-like pattern, microcalcifications, structural distortion and disruption, and ductal dilatation or thickening within the gland. According to the distribution characteristics, the MRI images of non-mass breast cancer can be classified as follows: focal distribution with a hypoechoic area affecting no more than one quadrant; linear or segmental distribution; regional, multi-regional, or diffuse distribution; with internal enhancement patterns including homogeneous, heterogeneous, clustered, or ring-like clustering (26). Different diagnostic methods have their own advantages and limitations. MRI is the most sensitive technique for early breast cancer detection; however, it has a low specificity and is expensive (27). Ultrasound is suitable for cases presenting with hypoechoic areas, while mammography is more appropriate for cases exhibiting microcalcifications (26). The combination of ultrasound and mammography can increase the detection rate of non-mass breast cancer (28). Therefore, clinicians should select the appropriate diagnostic method based on the patient’s condition to enhance the detection rate of non-mass breast cancer and minimize the risk of misdiagnosis or missed diagnosis.

Currently, there is no clear consensus on the treatment of rectal metastasis from breast cancer. Chemotherapy and molecular targeted therapies are the main treatment modalities for rectal metastasis from breast cancer. Regarding the clinical benefits of surgery, only a few cases of rectal metastasis from breast cancer are diagnosed in the emergency setting, often due to complications such as bowel obstruction, bleeding, or perforation. However, there is no unified opinion on whether to prioritize the treatment of the primary tumor or the metastatic site in cases of colorectal metastasis. Current guidelines suggest that, in the case of urgent conditions such as bleeding, obstruction, or perforation, colorectal metastasis should be managed first, followed by treatment of the primary tumor, after thoroughly assessing the primary cancer (29). While surgery can provide certain survival benefits, it does not significantly prolong overall survival (30).

With respect to the prognosis of this case, the patient is currently undergoing HP-targeted therapy but has declined chemotherapy. Given the substantial tumor burden, monotherapy with targeted agents may be insufficient to achieve sustained disease control, raising concerns about the potential emergence of therapeutic resistance, multifocal metastatic spread, and an overall unfavorable prognosis. While existing evidence (31) has demonstrated that HP-targeted therapy yields significant clinical benefit in advanced HER2-positive breast cancer, with improvements in both progression-free survival and overall survival. However, in patients with a heavy disease burden, the therapeutic effect may be limited. In such cases, upon disease progression following first-line targeted therapy, escalation to second-line regimens such as trastuzumab deruxtecan should be considered. Brain metastases are reported in approximately 50% of patients with advanced HER2-positive breast cancer (2), and represent a major clinical challenge that significantly impacts prognosis and quality of life. In case of brain metastasis from breast cancer, reasonable local treatment (radiotherapy or surgery) and supportive therapy should be selected based on the patient’s general condition, control of extracranial lesions, number and location of brain metastases, occupying effect, and risk of surgery. For systemic management, agents capable of crossing the blood–brain barrier, including tyrosine kinase inhibitors and antibody-drug conjugates, are generally preferred. Accordingly, during ongoing dual HP-targeted therapy, periodic evaluation of therapeutic response is essential to assess clinical benefit and inform timely adjustments to the treatment strategy.

The diagnosis and treatment of metastasis to rare sites remain limited due to the lack of comprehensive evidence. Clinicians typically rely on a small number of case reports and case series to inform treatment decisions (32). Currently, there are no specific guidelines or consensus on the management of rectal metastasis from breast cancer. We aim to actively publish cases with clear documentation of rare metastatic sites to enhance understanding of rare metastasis pathways in breast cancer and provide insights for future research.

## 4 Conclusion

In this case, the non-specific symptoms challenged the early diagnosis of breast cancer, highlighting the importance of women’s self-breast health awareness and breast cancer screening programs for the early diagnosis and treatment of breast cancer, and encouraging women to be proactive in observing any unusual breast changes and to seek medical attention in a timely manner. The uniqueness of this case in terms of metastatic site, breast cancer type, and clinical presentation provides ideas for future research.

## Data Availability

The original contributions presented in the study are included in the article/supplementary material. Further inquiries can be directed to the corresponding author/s.
